# Obstetric consequences of a false‐positive diagnosis of large‐for‐gestational‐age fetus

**DOI:** 10.1002/ijgo.14047

**Published:** 2021-12-09

**Authors:** Marta Papaccio, Anna Fichera, Alessia Nava, Sonia Zatti, Vera Gerosa, Federico Ferrari, Enrico Sartori, Federico Prefumo, Nicola Fratelli

**Affiliations:** ^1^ Division of Obstetrics and Gynecology ASST Spedali Civili and Department of Clinical and Experimental Sciences University of Brescia Brescia Italy

**Keywords:** birth weight, cesarean section, estimated fetal weight, large for gestational age, ultrasound prediction

## Abstract

**Objective:**

To compare delivery outcomes between true‐positive (TP) and false‐positive (FP) large‐for‐gestational‐age (LGA) fetuses, appropriate‐for‐gestational‐age (AGA) fetuses, and false‐negative (FN) LGA fetuses.

**Methods:**

Retrospective cohort study of singleton pregnancies at risk for macrosomia without contraindication to vaginal delivery, receiving an ultrasound scan at 34–37 weeks of pregnancy.

**Results:**

In all, 430 pregnancies were included: 155 TP LGA, 87 FP LGA, 177 AGA and 11 FN LGA newborns. Cesarean section rate during labor was significantly higher in FP LGA than in AGA (19% vs. 8.7%) but not significantly different between FP LGA and TP LGA (19% vs. 32.4%). Median birth weight *z* score was significantly higher in TP LGA (1.9) compared with the FP LGA and AGA (0.91 and 0.84, respectively), whereas no significant differences were found between FP LGA and AGA. Admission to a neonatal intensive care unit was significantly more frequent in TP LGA than AGA, whereas shoulder dystocia, postpartum hemorrhage, and third‐ to fourth‐degree perineal tears were similar between the different groups.

**Conclusion:**

A false‐positive diagnosis of LGA fetus is associated with a significant increase of cesarean section during labor. Therefore, a suspicious ultrasound may result in reduction of the clinical threshold for the diagnosis of abnormal labor.

## Abstract 2

A false‐positive diagnosis of LGA fetus on ultrasound at 34–37 weeks is associated with a significant increase in cesarean section during labor.

## 1 INTRODUCTION

The American College of Obstetricians and Gynecologists (ACOG), describes large for gestational age (LGA) as a birth weight equal to or greater than the 90th centile for a given gestational age, while macrosomia, whose definition is more controversial, is described as a birth weight over 4000 or 4500 g, unrelated to gestational age.^1^

A variety of maternal factors predispose to high infant birth weight: pre‐existing diabetes or gestational diabetes, pre‐pregnancy obesity, gestational weight gain, high birth weight in previous pregnancies, multiparity and ethnicity.^1^ LGA fetuses are associated with higher incidence of adverse perinatal outcome. Maternal complications include prolonged labor, labor augmentation with oxytocin, instrumental delivery, cesarean section, postpartum hemorrhage, and pelvic floor damage with development of anal/urinary stress incontinence and uterovaginal prolapse. Neonatal complications include shoulder dystocia and associated brachial plexus injury, fractured clavicle or humerus, meconium aspiration, hypoglycemia, perinatal asphyxia, and fetal death.^1^ LGA infants are also at increased risk of long‐term poor outcomes, such as obesity, type 2 diabetes mellitus, asthma, early cardiovascular disease, and increased future risk of certain types of cancer.^2^, ^3^, ^4^

Prenatal diagnosis could be useful to predict these complications, but ultrasound can be inaccurate to correctly identify LGA neonates. There are several models for estimation of fetal weight but the most widely accepted and accurate is the one published by Hadlock et al. in 1985,^5^ which combines ultrasonographic measurements of fetal abdominal circumference (AC), head circumference (HC) and femur length (FL) in the formula: Log_10_ (weight) = 1.326 − 0.00326 × AC × FL + 0.0107 × HC + 0.0438 × AC. Measurement errors in fetal biometry cause substantial error in estimated fetal weight, resulting in misclassification of LGA fetuses, which can lead to inappropriate clinical management.^6^ In fact, a false‐positive diagnosis of LGA fetuses may bias the intrapartum management practice, increasing the risk of cesarean section in labor.

The aim of the present study was to compare delivery outcomes between pregnancies with a false‐positive prenatal diagnosis of LGA fetus, true‐positive LGA and appropriate for gestational age (AGA) fetuses.

## 2 MATERIALS AND METHODS

### 2.1 Study design

This was a retrospective cohort study of singleton pregnancies at risk for macrosomia without contraindications to vaginal delivery, receiving an ultrasound scan between 34 and 37 weeks of pregnancy in a dedicated clinic between March 2011 and February 2018. Women were referred in case of: gestational diabetes; maternal body mass index (BMI; calculated as weight in kg divided by the square of height in m) greater than 30; or suspected LGA at the routine third‐trimester scan performed at 28–32 weeks of pregnancy according to national guidelines. Singleton pregnancies with gestational age confirmed by a first‐trimester scan and without absolute contraindications to vaginal delivery were included. The exclusion criteria were twin pregnancies, fetal malformations, unknown gestational age, previous cesarean section, and any contraindications to labor induction or vaginal delivery. Data were retrieved from medical records and from an electronic database (Viewpoint version 5, GE Healthcare, Munich, Germany) in which information about pregnancy, ultrasound examinations, labor, and postpartum and neonatal outcomes is collected. As per national regulations, the analysis of anonymized routinely collected clinical data did not require ethics committee approval. Written consent was obtained from all women.

Patients were divided into four groups based on estimated fetal weight (EFW) (based on the Hadlock et al. chart^5^) or AC (based on the Snijders et al. chart^7^), and birth weight (based on the Yudkin et al. chart^8^). False‐positive LGA (FP LGA) included fetuses with EFW or AC above the 90th centile and birth weight below the 90th centile; true‐positive LGA (TP LGA) included fetuses with EFW or AC above the 90th centile and birth weight above the 90th centile, and appropriate for gestational age (AGA) included fetuses with AC, EFW, and birth weight between the 50th and 90th centiles. False‐negative LGA (FN LGA) included fetuses with EFW and AC below the 90th centile and birth weight above the 90th centile.

Data analyzed were onset of labor (spontaneous versus induction), oxytocin augmentation, epidural analgesia, duration of labor, type of delivery (International Classification of Diseases ninth revision‐9 codes were used to identify indications for cesarean section^9^), third‐ and fourth‐degree perineal tear, postpartum hemorrhage (defined as blood loss >1000 ml), shoulder dystocia not resolved by McRoberts' maneuver, birth weight, umbilical cord arterial pH < 7, and admission to neonatal intensive care unit.

### 2.2 Statistical analysis

Median and interquartile range were used in descriptive statistics. The values of neonatal biometric variables were transformed into *z* scores to make them independent of gestational age. Continuous variables were compared by Mann‐Whitney *U* test or Kruskal‐Wallis test for the comparison between more than two groups. Dunn's test with Bonferroni correction was used to identify which specific group was different from the others.

Nominal variables were compared by Fisher exact test (comparison of two or more dichotomous variables and small samples) or χ^2^ test (sufficiently large samples, no contingency table cell with a value less than 5) with one or two degrees of freedom. Values of *P* less than 0.05 were considered statistically significant. stata 13.1 software was used for statistical analyses (StataCorp., College Station, TX, USA). We estimated that in order to achieve a power of 80% for detecting a difference in proportions of 0.20 in cesarean section rate between two groups at a two sided *P* value of 0.05, with an estimated 0.10 cesarean section rate in the AGA group, a sample size of *n* = 69 per group would be required.

## 3 RESULTS

A total of 430 patients were included in the study, with 155 TP LGA, 87 FP LGA, 177 AGA, and 11 FN LGA fetuses. The characteristics of the study population are shown in Table 1: ethnicity, parity, incidence of gestational diabetes, labor augmentation with oxytocin, and epidural analgesia were similar in the four groups. On the other hand, BMI at delivery, gestational age at ultrasound, and EFW had significant differences between groups. Induction of labor was significantly more frequent in TP LGA (57.0%) and FP LGA (28.2%) compared with the other groups.

**TABLE 1:** Characteristics of the study population^a^

	TP LGA (*n* = 155)	FP LGA (*n* = 87)	TN AGA (*n* = 177)	FN LGA (*n* = 11)	*P* value
BMI at delivery	29.1 (26.2–33.8)	29.7 (26.5–33.2)	27.8 (25.4–31.8)	27.73 (27.5–31.2)	0.031^b^
Ethnicity					
Caucasian	137 (35.9%)	76 (19.9%)	159 (41.6%)	10 (2.6%)	0.916
Black	3 (30%)	4 (40%)	3 (30%)	0	
Asian	11 (36.67%)	6 (20%)	12 (40%)	1 (3.3%)	
East Asian	3 (50%)	1 (16.7%)	2 (33.3%)	0	
Nulliparous	60 (38.7%)	39 (44.8%)	89 (50.28%)	5 (45.5%)	0.214
Gestational diabetes	46 (29.7%)	30 (34.5%)	43 (24.3%)	3 (27.3%)	0.348
Pre‐gestational diabetes	19 (12.3%)	3 (3.5%)	2 (1.13%)	0	<0.001^c^
Induction of labor	77 (57%)	38 (28.2%)	15 (11.1%)	5 (3.7%)	<0.001^d^
Oxytocin augmentation	10 (6.5%)	8 (9.2%)	21 (11.9%)	0	0.245
Epidural analgesia	33 (21.3%)	48 (55.2%)	58 (32.7%)	2 (18.2%)	<0.001^e^
EFW, g	3603 (3278–3939)	3288 (3053–3653)	3093 (2920–3321)	3630 (3249–4070)	0.001^f^
EFW, *z* score	0.70 (–0.06 to 1.43)	1.47 (1.32–1.64)	0.35 (–0.046 to 0.717)	0.46 (–0.68 to 1.03)	0.001^g^
Gestational age at ultrasound, week	36.0 (35.0–37.4)	35.7 (34.9–37.4)	36.8 (36–37.8)	37.1 (35.4–39.7)	0.001^h^

Abbreviations: BMI, body mass index (calculated as weight in kilograms divided by the square of height in meters); EFW, estimated fetal weight; FN LGA, false‐negative large for gestational age; FP LGA, false‐positive large for gestational age; TN AGA, true‐negative appropriate for gestational age; TP LGA, true‐positive large for gestational age.

^a^ Data expressed as number (percentage) or as median (interquartile range).

^b^ BMI at delivery: no significant difference between TP LGA and FP LGA group (*P* = 1); significant difference between TP LGA group and TN AGA group (*P* < 0.036); no significant difference between TP LGA and FN LGA group (*P* = 1); significant difference between FP LGA group and TN AGA group (*P* = 0.049) no significant difference between FP LGA group and FN LGA group (*P* = 1); no significant difference between TN AGA group and FN LGA group (*P* = 1).

^c^ Pregestational diabetes: significant difference between TP LGA and FP LGA group (*P* = 0.021); significant difference between TP LGA group and TN AGA group (*P* < 0.001); no significant difference between TP LGA and FN LGA group (*P* = 0.617); no significant difference between FP LGA group and TN AGA group (*P* = 0.335); no significant difference between FP LGA group and FN LGA group (*P* = 1); no significant difference between TN AGA group and FN LGA group (*P* = 1).

^d^ Induction of labor: no significant difference between TP LGA and FP LGA group (*P* = 0.370); significant difference between TP LGA group and TN AGA group (*P* < 0.001); no significant difference between TP LGA and FN LGA group (*P* = 1); significant difference between FP LGA group and TN AGA group (*P* < 0.001); no significant difference between FP LGA group and FN LGA group (*P* = 0.911); significant difference between TN AGA group and FN LGA group (*P* < 0.001).

^e^ Epidural analgesia: no significant difference between TP LGA and FP LGA group (*P* = 1); significant difference between TP LGA group and TN AGA group (*P* < 0.001); no significant difference between TP LGA and FN LGA group (*P* = 0.28); significant difference between FP LGA group and TN AGA group (*P* < 0.001); significant difference between FP LGA group and FN LGA group (*P* = 0.026); no significant difference between TN AGA group and FN LGA group (*P* = 0.507).

^f^ EFW (g): significant difference between TP LGA and FP LGA group (*P* = 0.002); significant difference between TP LGA group and TN AGA group (*P* < 0.001); no significant difference between TP LGA and FN LGA group (*P* = 1); significant difference between FP LGA group and TN AGA group (*P* < 0.001); no significant difference between FP LGA group and FN LGA group (*P* = 0.164); significant difference between TN AGA group and FN LGA group (*P*  < 0.001).

^g^ EFW (*z* score): significant difference between TP LGA and FP LGA group (*P* < 0.001); significant difference between TP LGA group and TN AGA group (*P* < 0.001); no significant difference between TP LGA and FN LGA group (*P* = 0.228); significant difference between FP LGA group and TN AGA group (*P* < 0.001); significant difference between FP LGA group and FN LGA group (*P* < 0.001); no significant difference between TN AGA group and FN LGA group (*P* = 1).

^h^ Gestational age at ultrasound: no significant difference between TP LGA and FP LGA group (*P* = 1); significant difference between TP LGA group and TN AGA group (*P* < 0.001); no significant difference between TP LGA and FN LGA group (*P* = 0.28); significant difference between FP LGA group and TN AGA group (*P* < 0.001); no significant difference between FP LGA group and FN LGA group (*P* = 0.170); no significant difference between TN AGA group and FN LGA group (*P* = 1).

**FIGURE 1:**
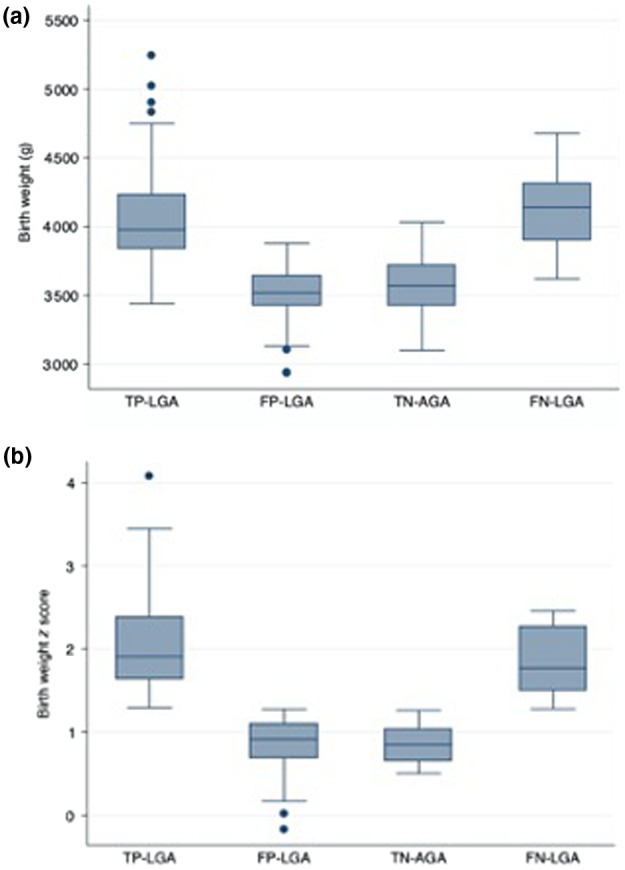
Birth weight (a) and birth weight *z* score (b) in the four groups. Abbreviations: FN LGA, false‐negative large for gestational age; FP LGA, false‐positive large for gestational age; TN AGA, true‐negative appropriate for gestational age; TP LGA, true‐positive large for gestational age. Birth weight was significantly higher in the TP LGA group compared with the false positive large for gestational age FP LGA (*P* < 0.001) and TN AGA (*P* < 0.001) groups; and in the FN LGA group compared to FP LGA (*P* < 0.001) and TN AGA groups (*P* < 0.001). No significant differences were found between the FP LGA and TN AGA groups (*P* = 0.322) and between TP LGA and FN LGA groups (*P* = 1). Birth weight *z* score was significantly higher in the TP LGA group compared with the FP LGA (*P* < 0.001) and TN AGA (*P* < 0.001) groups; and in the FN LGA group compared with the FP LGA (*P* < 0.001) and TN AGA (*P* < 0.001) groups. No significant differences were found between the FP LGA and TN AGA groups (*P* = 1) and between the TP LGA and FN LGA groups (*P* = 1)

Details on delivery shown in Table 2. The duration of labor and the incidence of operative vaginal delivery were similar in the four groups. The number of cesarean sections performed before labor was significantly higher in the TP LGA group. Moreover, there were significantly more cesarean sections performed in labor in the TP LGA and FP LGA groups (relative risk [RR] 3.7, 95% confidence interval [CI] 2.1–6.7 and RR 2.2, 95% CI 1.1–4.5, respectively, using AGA as the reference group). Birth weight was significantly higher in the TP LGA and FN LGA groups, whereas no significant difference was found between the FP LGA and AGA groups (Figure 1). Despite these findings, the cesarean section rate during labor was not significantly different between the FP LGA and TP LGA groups but the percentage was significantly higher in the FP LGA than in the AGA group. There were no significant differences for cesarean sections performed during induced labor, and rates of cesarean section performed in labor for dystocia were comparable among groups (Table 3).

**TABLE 2:** Delivery details^a^

	TP LGA (*n* = 155)	FP LGA (*n* = 87)	TN AGA (*n* = 177)	FN LGA (*n* = 11)		*P* value	
Gestational age at delivery, wk	38.4 (38–39)	38.7(38.1–39.3)	39.1 (38.4–40.1)		39.2 (38.7–40)		0.001^b^
Birth weight, g	3978 (3840–4230)	3520 (3430–3640)	3570 (3428–3720)		4140 (3900–4310)		0.001^c^
Birth weight (*z* score)	1.9 (1.64–2.39)	0.91 (0.68–1.1)	0.84 (0.66–1.03)		1.76 (1.5–2.27)		0.001^d^
CS not in labor	53 (34.1%)	20 (23%)	28 (15.8%)		4 (36%)		0.001^e^
Labor	102 (65.1%)	67 (77%)	149 (84.1%)		7 (64%)		
Spontaneous vaginal delivery	65 (63.7%)	51 (76%)	125 (83.9%)		6 (86%)		0.003^f^
Operative vaginal delivery	4 (3.9%)	3 (5%)	11 (7.4%)		1 (14%)		0.366
CS in labor	33 (32.4%)	13 (19%)	13 (8.7%)		0 (0%)		<0.001^g^
Duration of labor, min	240 (180–353)	240 (180–360)	240 (180–360)		177 (60–240)		0.316
Duration of I stage, min	210 (130–292.5)	203 (150–332)	225 (151–318)		149 (58–195)		0.293
Duration of II stage (min)	27 (13–55)	21 (13–50)	29 (13–60)		30 (10–42)		0.771

Abbreviations: CS, cesarean section; FN LGA, false‐negative large for gestational age; FP LGA, false‐positive large for gestational age; TN AGA, true‐negative appropriate for gestational age; TP LGA, true‐positive large for gestational age.

^a^ Data expressed as number (percentage) or as median (interquartile range).

^b^ Gestational age at delivery: no significant difference between TP LGA and FP LGA group (*P* = 0.175); significant difference between TP LGA group and TN AGA group (*P* < 0.001); significant difference between TP LGA and FN LGA group (*P* = 0.004); significant difference between FP LGA group and TN AGA group (*P* = 0.001); no significant difference between FP LGA group and FN LGA group (*P* = 0.055); no significant difference between TN AGA group and FN LGA group (*P* = 1).

^c^ Birth weight: significant difference between TP LGA and FP LGA group (*P* < 0.001); significant difference between TP LGA group and TN AGA group (*P* < 0.001); no significant difference between TP LGA and FN LGA group (*P* = 1); no significant difference between FP LGA group and TN AGA group (*P* = 0.322) significant difference between FP LGA group and FN LGA group (*P* < 0.001); significant difference between TN AGA group and FN LGA group (*P* < 0.001).

^d^ Birth weight *z* scores: significant difference between TP LGA and FP LGA group (*P* < 0.001); significant difference between TP LGA group and TN AGA group (*P* < 0.001); no significant difference between TP LGA and FN LGA group (*P* = 1); no significant difference between FP LGA group and TN AGA group (*P* = 1); significant difference between FP LGA group and FN LGA group (*P* < 0.001); significant difference between TN AGA group and FN LGA group (*P* < 0.001).

^e^ CS not in labor: no significant difference between TP LGA and FP LGA group (*P* = 0.068); significant difference between TP LGA group and TN AGA group (*P* < 0.001); no significant difference between TP LGA and FN LGA group (*P* = 1); no significant difference between FP LGA group and TN AGA group (*P* = 0.156); no significant difference between FP LGA group and FN LGA group (*P* = 0.455); no significant difference between TN AGA group and FN LGA group (*P* = 0.095).

^f^ Spontaneous vaginal delivery: no significant difference between TP LGA and FP LGA group (*P* = 0.089); significant difference between TP LGA group and TN AGA group (*P* < 0.001); no significant difference between TP LGA and FN LGA group (*P* = 0.418); no significant difference between FP LGA group and TN AGA group (*P* = 0.174); no significant difference between FP LGA group and FN LGA group (*P* = 1); no significant difference between TN AGA group and FN LGA group (*P* = 1).

^g^ CS in labor: no significant difference between TP LGA and FP LGA group (*P* = 0.064); significant difference between TP LGA group and TN AGA group (*P* < 0.001); no significant difference between TP LGA and FN LGA group (*P* = 0.099); significant difference between FP LGA group and TN AGA group (*P* = 0.04); no significant difference between FP LGA group and FN LGA group (*P* = 0.341); no significant difference between TN AGA group and FN LGA group (*P* = 1).

**TABLE 3:** Cesarean sections performed in labor^a^

	TP LGA	FP LGA	TN AGA	FN LGA	*P* value
CS in labor	33/102 (30%)	13/67(21%)	13/149 (9%)	0/7	<0.001^b^
CS in induced labor	24/75(32%)	5/35 (14%)	2/15 (13%)	0/5	0.072
CS in spontaneous labor	9/27 (33%)	8/32 (25%)	11/134 (8%)	0/2	0.001^c^
CS for dystocia (in total number of CS in labor)	11/33 (33%)	6/13 (46%)	8/13 (62%)	0/0	0.2
CS for dystocia (in total number of labors)	11/102 (10%)	6/67 (10%)	8/149 (5%)	0/7	0.356

Abbreviations: CS, cesarean section; FN LGA, false‐negative large for gestational age; FP LGA, false‐positive large for gestational age; TN AGA, true‐negative appropriate for gestational age; TP LGA, true‐positive large for gestational age.

^a^ Data expressed as number (percentage).

^b^ CS in labor: no significant difference between TP LGA and FP LGA group (*P* = 0.064); significant difference between TP LGA group and TN AGA group (*P* < 0.001); no significant difference between TP LGA and FN LGA group (*P* = 0.099); significant difference between FP LGA group and TN AGA group (*P* = 0.04); no significant difference between FP LGA group and FN LGA group (*P* = 0.341); no significant difference between TN AGA group and FN LGA group (*P* = 1).

^c^ CS in spontaneous labor: no significant difference between TP LGA and FP LGA group (*P* = 0.481); significant difference between TP LGA group and TN AGA group (*P* < 0.001); no significant difference between TP LGA and FN LGA group (*P* = 0.1); significant difference between FP LGA group and TN AGA group (*P* = 0.007); no significant difference between FP LGA group and FN LGA group (*P* = 1); no significant difference between TN AGA group and FN LGA group (*P* = 1).

As shown in Table 4, BMI at delivery, induction of labor, and gestational age at delivery, which was significantly different between the groups, were not associated with a higher incidence of cesarean section in labor. Instead, pregnancies with prenatal suspicion of LGA fetus (TP LGA or FP LGA) had an increased risk of cesarean section during labor, of 5.9‐fold in TP LGA and 2.6‐fold in FP LGA. Table 5 shows the remaining delivery outcomes.

**TABLE 4:** Factors associated with cesarean section in labor^a^

	aOR (95% CI)	*P* value
BMI at delivery	1.03 (0.97–1.10)	0.309
Gestational age at delivery	0.93 (0.67–1.30)	0.684
Induction of labor	0.77 (0.36–1.64)	0.496
FP LGA	2.63 (1.05–6.6)	0.039
TP LGA	5.9 (2.3–15.1)	<0.001
Constant	0.606 (1.04e–06–355168)	0.941

No correlations were found between BMI at delivery, gestational age at delivery, induction of labor and cesarean section performed in labor. FP LGA and TP LGA fetuses had an increased risk of cesarean section in labor when compared with appropriate for gestational age fetuses (taken as reference, aOR = 1).

Abbreviations: aOR, adjusted odds ratio; BMI, body mass index (calculated as weight in kg divided by the square of height in m); CI, confidence interval; FP LGA, false‐positive large for gestational age; TP LGA, true‐positive large for gestational age.

^a^ Adjusted risks of cesarean section in labor assessed by logistic regression and aOR with 95% CI in brackets are shown. The dependent variable is the cesarean section in labor. The aOR take into account the effect of BMI at delivery, gestational age at delivery, induction of labor, and true or false diagnosis of LGA.

**TABLE 5:** Secondary outcomes^a^

	TP LGA (*n* = 155)	FP LGA (*n* = 87)	TN AGA (*n* = 177)	FN LGA (*n* = 11)	*P* value
Shoulder dystocia	2 (1.29%)	0 (0%)	0 (0%)	1 (9%)	0.023^b^
Third‐ to fourth‐degree perineal tear	1 (0.65%)	1 (1.15%)	2 (1.13%)	0	1
PPH > 1000 ml	24 (15.5%)	8 (9.2%)	22 (12.4%)	3 (27%)	0.241
Umbilical cord arterial pH < 7.0	0 (0%)	2 (2.3%)	1 (0.6%)	0	0.224
Umbilical cord arterial pH < 7.1	6 (3.87%)	4 (4.6%)	6 (3.39%)	1 (9.09%)	0.590
Umbilical cord arterial pH < 7.2	32 (20.65%)	20 (22.99%)	32 (18.08%)	3 (27.27%)	0.666
Admission to NICU	7 (4.52%)	3 (3.54%)	1 (0.6%)	1 (9%)	0.037^c^

Abbreviations: FN LGA, false‐negative large for gestational age; FP LGA, false‐positive large for gestational age; NICU, neonatal intensive care; PPH, postpartum hemorrhage;TN AGA, true‐negative appropriate for gestational age; TP LGA, true‐positive large for gestational age.

^a^ Data expressed as number (percentage).

^b^ Shoulder dystocia: no significant difference between TP LGA and FP LGA group (*P* = 0.538); no significant difference between TP LGA group and TN AGA group (*P* = 0.217); no significant difference between TP LGA and FN LGA group (*P* = 0.187); no significant difference between FP LGA group and FN LGA group (*P* = 0.112); no significant difference between TN AGA group and FN LGA group (*P* = 0.059).

^c^ Admission to NICU: no significant difference between TP LGA and FP LGA group (*P* = 1); significant difference between TP LGA group and TN AGA group (*P* = 0.028); no significant difference between TP LGA and FN LGA group (*P* = 0.429); no significant difference between FP LGA group and TN AGA group (*P* = 0.106); no significant difference between FP LGA group and FN LGA group (*P* = 0.384); no significant difference between TN AGA group and FN LGA group (*P* = 0.114).

## 4 DISCUSSION

We showed that a false‐positive diagnosis of LGA fetus can have clinical consequences: the incidence of cesarean section performed in labor in the FP LGA group was more than twice the AGA group (19% vs. 8.7%), whereas it was comparable to the incidence in the TP LGA group (32.4%).

In the AGA group we included fetuses with EWF, cesarean section, and birth weight between the 50th and 90th centiles rather than the most commonly used range of 10th to 90th centiles, as usual. We selected the larger within the appropriately grown fetuses in order to make the sample more comparable with the other two groups in terms of maternal and neonatal complications.

Pregnancies complicated by fetal macrosomia have an increased incidence of complications in labor, and in particular a greater risk of cesarean section.^10^ However, in the present study population the increased incidence of cesarean section in labor in the FP LGA group compared with the AGA group did not correspond to a difference in birth weight, either in absolute terms or in terms of weight centiles by gestational age (Figure 1). The comparable incidence of cesarean section in labor between FP LGA and TP LGA was associated with a significantly higher birth weight in TP LGA fetuses. Furthermore, in accordance with published data,^11^ there was no difference in the duration of labor between TP LGA, FP LGA, FN LGA, and AGA fetuses.

In our unit, when an LGA fetus is suspected by ultrasound at 34–37 weeks of pregnancy, in the absence of contraindications to vaginal delivery, induction of labor is offered at 37–39 gestational weeks to reduce the maternal and neonatal risks associated with macrosomia.^12^, ^13^ Despite a higher incidence of induced labor in the FP LGA group compared with the AGA group (see Table 1), in our cohort there was no significant difference in the incidence of cesarean sections performed in labor after induction between these groups (Table 3). The higher incidence of induction of labor resulted in a lower gestational age at birth in the FP LGA group compared with the AGA group.

Induction of labor, gestational age at delivery and BMI at delivery, a known risk factor for macrosomic and LGA fetuses,^1^, ^14^, ^15^, ^16^ were differently distributed between the three groups but did not significantly influence the risk of cesarean section in labor (odds ratio [OR] 0.77, OR 0.93 and OR 1.03, respectively; Table 4). Prenatal suspicion of an LGA fetus was related to an increased incidence of cesarean section in labor of 5.9‐fold in TP LGA and 2.63‐fold in FP LGA.

Unfortunately, the relatively small size of our sample and the design of the study do not allow evaluation of the contribution of the different indications (International Classification of Diseases ninth revision codes)^9^ in the increase of cesarean section in the FP LGA group, even if dystocia in labor appeared to be comparable between the three groups.

Our results are in agreement with several previous studies. Melamed et al.,^17^ Blackwell et al.,^11^ and Pretscher et al.^18^ evaluated the potential consequences of an incorrect diagnosis of fetal macrosomia on the course of labor in retrospective studies, in which they demonstrated that the probability of cesarean section in labor increases from two‐ to five‐fold in case of overestimation of fetal weight, regardless of birth weight. Levine et al.^19^ assessed whether the diagnosis of an LGA fetus in the third trimester was associated with different management of labor and delivery, demonstrating a significant increase in the diagnosis of labor abnormalities and in performing elective cesarean sections in women with a fetus diagnosed as LGA.

In such studies the ultrasound scan for the estimation of fetal weight was carried out shortly before delivery at term of pregnancy.^11^, ^17^, ^18^, ^19^ On the contrary, in the present study the estimation of fetal weight in pregnancies at risk for macrosomia was carried out between the 34th and the 37th weeks of gestation, when the estimation can be made with greater accuracy.^15^, ^19^ At the end of pregnancy, the resolution of ultrasound and the accuracy of measurements decrease because of the reduction in amniotic fluid, the greater calcification of fetal bones, and the position of the fetal head lower in the pelvis.

However, our data confirm the inaccuracy of ultrasound in the identification of LGA. Although ultrasound scans were performed at the best timing, about 36 weeks of pregnancy,^15^, ^20^, ^21^ we had a 20% false‐positive rate (82 FP LGA in 251 fetuses estimated LGA). Possible explanations for the modest performance of EFW include that relatively small errors in the measurements of fetal HC, CA, and FL could have significant impact on EFW^6^; moreover, the fetal growth curve can change over time between ultrasound and delivery. Various efforts were made to improve the accuracy in estimation of fetal weight, but also the customized growth curves do not seem to perform better than population‐based growth curves in the diagnosis of increased fetal growth or in the prediction of associated complications.^22^, ^23^ Nevertheless, these data possibly influence the diagnosis of false positives for LGA, but not the consequences in clinical management.

The other complications analyzed (postpartum hemorrhage, third‐ to fourth‐degree perineal tear and shoulder dystocia) had comparable results in the three groups. As these are rare events, a larger sample would be needed to evaluate the real association with the diagnosis of TP LGA and FP LGA. In particular, shoulder dystocia, the feared complication associated with significant perinatal morbidity and mortality, has an incidence between 0.58% and 0.70%. We have excluded from the study the cases of shoulder dystocia resolved by McRoberts' maneuver, which consists in flexion and abduction of the maternal thighs on the abdomen. This technique is the least invasive planned maneuver and the first intervention that should be performed, with reported success rates of about 90%.^24^

Finally, the only secondary complication that with significantly different results among the three groups was neonatal intensive care unit admission.

One main limitation of the present study is its retrospective nature; data were retrieved from medical records and from an electronic database employed to collect gestational and perinatal information. Moreover, the sample size was relatively small. This did not allow us to evaluate the contribution of the different indications in the increase of cesarean section in the FP LGA group, even if dystocia in labor appeared to be comparable between the three groups. A larger sample would be needed to evaluate the real association between the diagnosis of TP LGA and FP LGA and postpartum hemorrhage, third‐ to fourth‐degree perineal tear and shoulder dystocia. Additionally, the small number of FN LGA fetuses precluded reliable comparisons of this group with the others. The main strength is that in our center the management of the suspected LGA fetuses, and consequently of the fetuses of both groups, TP LGA and FP LGA, is carried out according to a standardized and uniform protocol.

In conclusion, a false‐positive diagnosis of LGA fetus on ultrasound at 34–37 weeks is associated with a significant increase in cesarean section during labor. Therefore, a suspicious ultrasound may result in reduction of the clinical threshold for the diagnosis of abnormal labor.

## CONFLICTS OF INTEREST

The authors have no conflicts of interest.

## AUTHOR CONTRIBUTIONS

FP and NF conceived and designed this study. MP, AF, AN, SZ, and VG contributed substantially to the acquisition of the data. MP, AF, AN, SZ, VG, FF, ES, FP, and NF contributed to the interpretation of the results. MP, AF, FP, and NF drafted the paper. MP, AF, AN, SZ, VG, FF, ES, FP, and NF revised and approved the final version of the manuscript.

## Data Availability

The data that support the findings of this study are available from the corresponding author upon reasonable request.
